# Perioperative changes in cardiac biomarkers in juvenile cats during neutering

**DOI:** 10.3389/fvets.2022.1008765

**Published:** 2022-10-04

**Authors:** Keisuke Konishi, Mei Sakamoto, Chikara Satake, Mitsuhiro Isaka, Seiji Okazaki, Shota Kono, Takayuki Nakamura, Hideki Tashiro, Takahiro Ushigusa

**Affiliations:** ^1^Yokohama Animal Medical Center Kannai Animal Clinic, Yokohama, Japan; ^2^FUJIFILM VET Systems Co., Ltd., Tokyo, Japan; ^3^Laboratory of Companion Animal Surgery, Department of Companion Animal Clinical Sciences, School of Veterinary Medicine, Rakuno Gakuen University, Hokkaido, Japan

**Keywords:** atrial natriuretic peptides, high-sensitive cardiac troponin I, juvenile cat, neutering surgery, perioperative myocardial injury

## Abstract

Perioperative myocardial injury (PMI) is commonly caused by myocardial ischemia that develops during or after non-cardiac surgery. It occurs in 17.9% of human patients after non-cardiac surgery due to elevated high-sensitive perioperation cardiac troponin. However, PMI has not been demonstrated in cats. To investigate its occurrence, this study aimed to analyze the perioperative changes in cardiac biomarkers and clinical data, including measurement of vital signs, echocardiography, blood pressure, electrocardiogram, X-ray, and anesthetic profile, in 30 juvenile cats under neutering surgery. All cats had increased high-sensitive cardiac troponin I (hs-cTnI) postsurgery compared with presurgery. In particular, 48% of cats (14/29) showed elevated hs-cTnI over a reference range after surgery. In all groups, hs-cTnI and systolic arterial blood pressure (SAP) were significantly higher at 0 h and 18 h postoperation than at preoperation. A significant positive correlation was found between hs-cTnI and SAP at 18 h postoperation. Atrial natriuretic peptides, heart rate, and left ventricular wall thickness were markedly higher at 0 h postoperation than at preoperation; however, respiratory rate and body temperature were significantly lower at 0 h postoperation than at preoperation. Anesthetic time and hs-cTnI were significantly higher at 18 h postoperation in females than in males. Significant positive correlations were observed between hs-cTnI and anesthetic time at 18 h postoperation in females. These results indicate that postoperative hs-cTnI level can greatly increase in juvenile cats and hs-cTnI measurement at perioperation is potentially beneficial for early detection and evaluation of the presence of PMI.

## Introduction

Perioperative myocardial injury (PMI) is commonly caused by myocardial ischemia that develops during or after non-cardiac surgery. It has recently been recognized as myocardial injury after non-cardiac surgery (MINS) in humans (1, 2). More than 80% of patients with MINS are asymptomatic and undetected, and MINS is associated with an ~10-fold increase in 30-day mortality (1). MINS is diagnosed when at least one postoperative cardiac troponin concentration exceeds the 99th percentile of the upper reference limit for the cardiac troponin assay as a consequence of a presumed ischemic event without evident non-ischemic causes (3). In humans, 17.9% of patients suffer from MINS due to elevated high-sensitive cardiac troponin (hs-cT) at perioperation (4). In dogs and cats, the definition of MINS has not been established. However, 55% of dogs have been reported to experience an increase in cardiac troponin I (cTnI) after general anesthesia (5). Conversely, no research has demonstrated the perioperative change in cTnI in cats.

Recently, transient myocardial thickening (TMT), characterized by a hypertrophic myocardiopathy (HCM) phenotype, has been described in cats. TMT has reverse remodeling with normalization of cardiac structure and function in contrast to progressive deterioration of cardiac function in HCM (6). TMT is relatively uncommon but predisposed in younger cats with an antecedent event such as general anesthesia for neutering. It is similar to stress-induced (takotsubo) cardiomyopathy in humans in terms of occurrence after antecedent stressful events including surgery (6, 7). Cardiac troponin is significantly elevated in TMT in cats and takotsubo cardiomyopathy, suggesting that myocardial injury occurs after antecedent stressful events (6, 8).

This study aimed to evaluate the perioperative changes in cardiac biomarkers in juvenile cats during neutering. Their changes were compared using anesthesia profile, electrocardiogram (ECG), blood pressure, X-ray, and echocardiographic characteristics to evaluate how surgery and anesthesia induce myocardial injury.

## Materials and methods

### Study design

Thirty intact client-owned cats, 15 males and 15 females, aged 5–13 months and weighing 2.0–4.55 kg were included in this study after obtaining informed consent from owners. All male cats, including two with deciduous tooth extractions and three with microchip insertions, underwent castration. All female cats, including seven with microchip insertions, underwent spaying (nine cats with ovariohysterectomy and six with ovariectomy). As a preoperative evaluation, no clinical signs and diseases were confirmed in all cats by clinical pathology examination including complete blood count and serum biochemistry exam (plasma protein concentration, renal and hepatic functions). All cats with cardiovascular diseases, such as HCM, as determined using echocardiography, thoracic radiography, ECG, and oscillometric indirect blood pressure measurement, were excluded from the study. Other exclusion criteria for cats were as follows: increased end-diastolic left ventricular wall thickness (LVWT ≥ 6 mm) and dilated left atrial [left atrium to aorta ratio (LA/Ao) ≥ 1.6] before surgery (9, 10). The cats with positive feline immunodeficiency virus (FIV) and/or feline leukemia virus (FeLV) in the past were excluded from this study, while FIV, FeLV, and feline coronavirus (FCoV) were not examined during this study. However, FIV and FeLV were negative at the time the cats first arrived at the owner's homes according to owners' reports and all cats in this study had lived indoors with no contact with FIV and/or FeLV carriers. The cats with clinical signs that suggested feline infectious peritonitis (FIP) were excluded from this study because there was a report of FCoV-associated myocarditis in FIP (11). This study was approved by the Ethics Committee for the Use of Animals in Research of Yokohama Animal Medical Center Kannai Animal Clinic, Yokohama, Kanagawa, Japan (Approval number: 2021-01; Approval date: September 15, 2021).

### Cardiac biomarker analysis

Venous blood samples were collected from the medial saphenous vein within 6 h before operation (pre) and at 0 h (post-0 h) and 18 h (post-18 h) after operation. All samples were separated and centrifuged in serum separation tubes and in plasma separation tubes containing aprotinin to measure high-sensitive cardiac troponin I (hs-cTnI) and atrial natriuretic peptide (ANP), respectively. The serums and plasmas were quickly stored in Eppendorf tubes at −20°C. These samples were immediately sent to the external laboratory (FUJIFILM VET Systems, Co., Ltd., Tokyo, Japan) in a frozen state. All serum concentrations of hs-cTnI and ANP in 27 cats were measured using a chemiluminescent immunoassay for detecting human cTnI (ADVIA Centaur CP TnI-ultra, Siemens Healthineers Japan, Tokyo, Japan) and human ANP (CL JACK determiner-CL ANP, Minaris Medical, Tokyo, Japan) within 3 days after blood sample collection. Each measurement range was 0.006–50.0 ng/mL [reference interval (RI): ≤ 0.121 ng/mL] in hs-cTnI and 10–2,000 pg/mL (RI: ≤ 102.7 pg/mL) in ANP. hs-cTnI and ANP values below the detection limit were assigned values of 0.006 ng/mL and 10 pg/mL, respectively, for the statistical analysis. Troponin is stable at −70 to −80°C, but not sufficient at −20°C for long-term storage (12). Short-term storage up to 3 months at −20°C is acceptable in cats (13). Thus, sample analysis was considered in this study because of the short-term storage (within 3 days) and immediate analysis at the external laboratory.

### Echocardiographic analysis

Echocardiographic video loops in all cats were recorded at three measurement points (pre, post-0 h, post-18 h) without sedation or stress-reducing drugs, such as gabapentin. The thickest end-diastolic left ventricular free wall and end-diastolic interventricular septal in three different cardiac cycles were measured in the right parasternal long-axis (RPLA) 4- or 5-chamber view and short-axis view (RPSA) at the papillary muscle level by using the leading-edge method, and the highest value among the averages of the three records in each view was defined as LVWT (6). The internal diameters of the left ventricle in diastole and systole were recorded from three different cardiac cycles in B-mode RPLA at chordae tendineae by using the inner-edge to inner-edge method, and the average of left ventricular fractional shortening (LVFS%) was calculated in B-mode RPLA (6, 14). LA/Ao and left atrial fractional shortening (LAFS%) at the heart base were measured from the B-mode RPSA view in the frame after aortic valve closure and the M-mode RPSA view, respectively (9). The left atrial diameter (LAD) was measured from the RPLA four-chamber view at end-systole (10). The presence or absence of systolic anterior motion of the mitral valve (SAM) and dynamic left ventricular outflow tract obstruction (DLVOTO) were assessed from the RPLA 5-chamber view and color Doppler (10).

### Anesthetic protocol

All cats underwent neutering surgery with the same preanesthetic and anesthetic drugs. After intravenous catheter was placed in the cephalic vein, the cats were premedicated with 0.02 mg/kg buprenorphine IV, 2 mg/kg robenacoxib SC, and 8 mg/kg cefovecin sodium SC. An atropine dose of 0.02 mg/kg was administered intravenously, and anesthesia was induced with 4 mg/kg alfaxalone IV to effect. Endotracheal tube was placed with administration of 0.4 mg lidocaine onto the arytenoid cartilages. After endotracheal intubation, 2–3% concentration of isoflurane with oxygen was maintained to effect. Intravenous crystalloid fluid was administered at rate of 3 mL/kg/h during operation. When hypotension [systolic arterial blood pressure (SAP) < 90 mmHg] developed, isoflurane concentration was reduced by SAP ≥ 90 mmHg. Anesthetic time was defined as from the administration of alfaxalone to extubation with regained consciousness, and post-0 h was defined as after extubation with regained consciousness.

### Clinical data

Clinical data including age, breed, sex, surgical procedure, and body weight (BW) of all cats were obtained. Moreover, vital signs including body temperature (BT), respiratory rate (RR), presence of a murmur or gallop, and heart rate (HR) calculated in echocardiography were recorded at three measurement points. Indirect blood pressure, ECG, and thoracic radiography were also recorded at three measurement points. Indirect blood pressure was measured using an oscillometric monitor (Bioscope A130; FUKUDA ME KOGYO, Co., Ltd., Tokyo, Japan). An appropriate size cuff was attached at the root of the tail in sternal recumbency after it settled down. At least seven readings were obtained from each cat at three measurement points, and the mean of five readings was calculated without the minimum and maximum SAP among the seven readings. Lead II ECG was acquired in right lateral recumbency using ECG monitoring and a recording device (Bioscope A130; FUKUDA ME KOGYO, Co., Ltd., Tokyo, Japan). Thoracic radiography in all cats was recorded to evaluate vertebral heart size (VHS).

### Statistical analysis

All data were tested for normality by using the Shapiro–Wilk test, and non-paired data were tested for homogeneity of variances by using the Levene's test. Repeated measures single-factor analysis of variance and the Friedman test were used to compare parametric and non-parametric data, respectively, among three measurement points. The Bonferroni test for parametric data and the Scheffe test for non-parametric data were used as *post-hoc* tests to determine between-group differences. Repeated measures two-way factorial analysis of variance for parametric data and the Mann–Whitney *U*-test for non-parametric data were used to test between-group differences. The Fishers exact test for categorical data was carried out to examine between-group differences. The correlations among hs-cTnI, ANP, and other variables were evaluated using the Spearman's rank correlation coefficient. Statistical significance was set at *P* < 0.05.

## Results

Thirty cats without cardiomyopathy met the inclusion criteria. The clinical characteristics of all cats are presented in Table 1. Most cats were pure-breds (23/30) including Ragdoll (4), American short hair (3), Munchkin (3), Scottish fold (3), Persian (2), Russian Blue (2), British short hair (1), Exotic short hair (1), Kinkalow (1), Maine Coon (1), Norwegian forest cat (1), and Ragamuffin (1). The remaining cats were non-purebred cats (7/30) (Domestic short hair). No significant difference was observed between breed and sex (Table 1). All cats were fully vaccinated with core vaccines for kittens, including feline panleukopenia virus, feline herpesvirus type-1, and feline calicivirus, before this study. No cats were vaccinated with the rabies vaccine because there is no legal requirement for cats in Japan. One cat had a history of roundworm infestation (*Toxocara cati*), but was already dewormed before this study. The remaining cats had no clinical signs that were suggestive of intestinal parasites, while 43% of cats (13/30) were treated monthly with preventatives, such as selamectin and sarolaner, for internal and external parasites (heartworm, helminths, ticks, ear mites, and fleas). No cats showed systemic hypertension (SAP > 160 mmHg) before surgery. No SAM or DLVOTO was found in echocardiography throughout the study. The mean anesthetic time was 45 (20–96) min in all groups, 32 (20–47) min in the male group, and 57 (42–96) min in the female group. Eight cats (27%) had hypotension (SAP < 90 mmHg) over 10 min during surgery and recovered to normotension (SAP ≥ 90 mmHg) within 15 min by adjusting isoflurane concentration. No significant difference was observed between the non-hypotension (*n* = 21) and hypotension (*n* = 8) groups during surgery in hs-cTnI (*P* = 0.558 at post-0 h, *P* = 0.143 at post-18 h) and ANP (*P* = 0.484 at post-0 h, *P* = 0.715 at post-18 h). No cats experienced hypertension (SAP > 180 mmHg), tachycardia (>240 beats per minute), and bradycardia (< 100 beats per minute) during surgery. One cat had hs-cTnI over RI (0.158 ng/mL, RI: ≤ 0.121 ng/mL) at pre without obvious abnormality of cardiac function and parameters. Fourteen out of 29 cats (48%) including 10 out of 14 females (71%) and four out of 15 males (27%) without hs-cTnI over reference range before surgery had excessive reference range of hs-cTnI at either post-0 h or post-18 h.

**Table 1:** Summary of clinical data before and during surgery in all cats.

	**Total (*n* = 30)**	**Male (*n* = 15)**	**Female (*n* = 15)**	***P*-value**
Breed (non-pure bred:purebred)	7 DSH, 4 Ragdoll, 3 ASH, 3 Munchkin, 3 Scottish fold, 2 Persian, 2 Russian Blue, 1 BSH, 1 ESH, 1 Kinkalow, 1 Maine Coon, 1 NFC, 1 Ragamuffin, (7:23)	2 ASH, 2 DSH, 2 Munchkin, 2 Ragdoll, 1 ESH, 1 Kinkalow, 1 Munchkin, 1 NFC, 1 Persian, 1 Ragamuffin, 1 Russian Blue, (2:13)	5 DSH, 2 Ragdoll, 2 Scottish fold, 1 ASH, 1 BSH, 1 Maine Coon, 1 Munchkin, 1 Persian, 1 Russian Blue, (5:10)	0.195
Body weight (kg)	3.05 (2.0–4.6)	3.7 (2.2–4.6)	2.9 (2.0–3.4)	0.010**
Age (month)	6 (5–13)	6 (5–13)	6 (5–12)	0.763
Murmur or gallop (%)	0/30 (0%)	0/15 (0%)	0/15 (0%)	–
Arrhythmia (%)	0/30 (0%)	0/15 (0%)	0/15 (0%)	–
Dehydration (%)	0/30 (0%)	0/15 (0%)	0/15 (0%)	–
Anesthetic time (min)	43 (20–96)	33 (20–47)	58 (42–96)	*P* < 0.001
Hypotension (SAP < 90 mmHg) during surgery (%)	8/30 (27%)	0/15 (0%)	8/15 (53%)	0.001**
Hypertension (SAP > 180 mmHg) during surgery (%)	0/30 (0%)	0/15 (0%)	0/15 (0%)	–
Tachycardia (>240 bpm) during surgery (%)	0/30 (0%)	0/30 (0%)	0/30 (0%)	–
Bradycardia (< 100 bpm) during surgery (%)	0/30 (0%)	0/30 (0%)	0/30 (0%)	–

Values are presented as mean for parametric data or median for non-parametric data. The values of body weight, age, and anesthetic time are presented as median because they are not normally distributed (non-parametric data). Asterisk indicates statistical difference between males and females (**P < 0.01). SAP, systolic arterial blood pressure; DSH, domestic short hair; ASH, American short hair; BSH, British short hair; ESH, exotic short hair; NFC, Norwegian forest cat; bpm, beats per minute.

**Figure 1:**
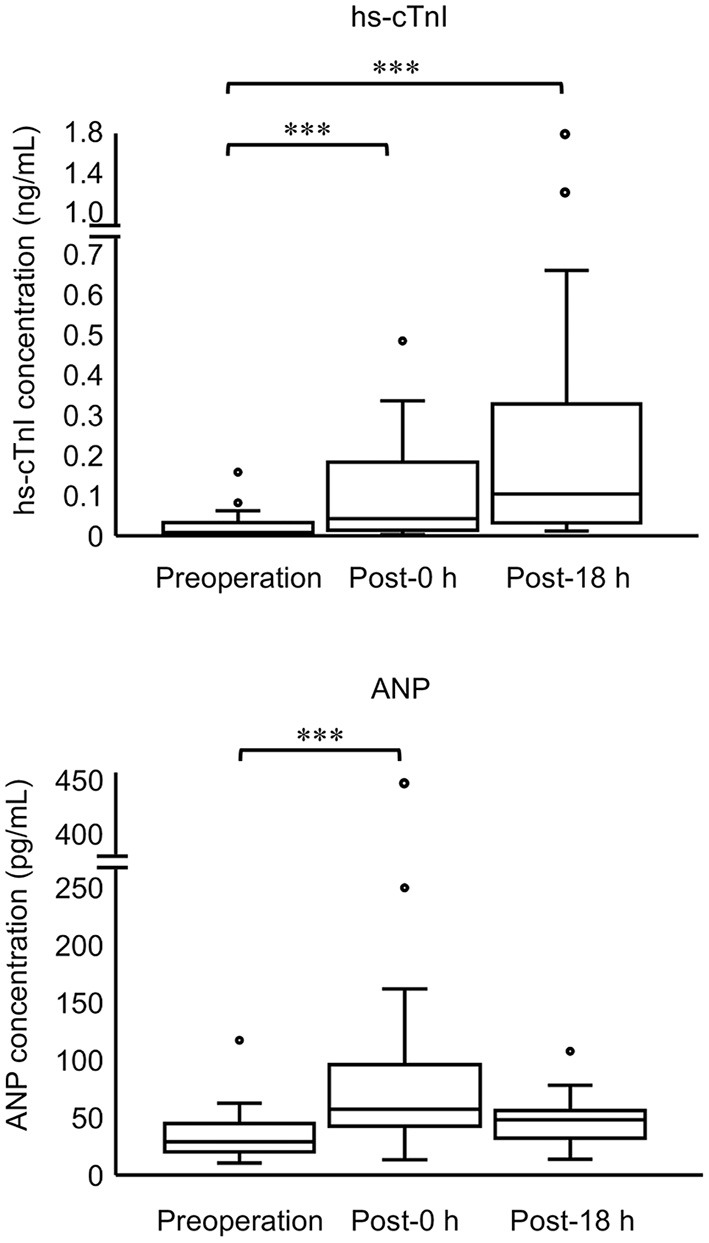
Concentrations of hs-cTnI and ANP in all cats at three measurement points (preoperation, 0 h postoperation, and 18 h postoperation). The top and bottom of the box represent the 75th and 25th percentiles, respectively; middle line, median; whiskers, highest and lowest data points within 1.5 times the length of the quartiles; and circles, outliers. Data of hs-cTnI and ANP are not normally distributed (non-parametric data). Asterisks indicate statistical difference (***P < 0.001).

Thirty cats (100%) had an increase in hs-cTnI at either post-0 h or post-18 h compared with pre. The levels of hs-cTnI and SAP were significantly higher at post-0 h and post-18 h than at pre (Figure 1; Table 2). The levels of ANP, HR, and LVWT were significantly higher at post-0 h than at pre, whereas the levels of RR and BT were significantly lower at post-0 h than at pre (Figure 1; Table 2). The levels of BT and HR at post-18 h significantly recovered from post-0 h to the level at pre (Table 2). No significant difference was noted in other variables among three measurement points (Table 2).

**Table 2:** Echocardiographic measurements and clinical data of all cats at pre-operation, 0 h post-operation, and 18 h post-operation.

**Variables**	**Pre vs. 0 h post**		**Pre vs. 18 h post**		**0 h post vs. 18 h post**	
	**Value**	***P*-value**	**Value**	***P*-value**	**Value**	***P*-value**
Body temperature (°C)	38.4 vs. 37.5	*P* < 0.001	38.4 vs. 38.5	0.844	37.5 vs. 38.5	*P* < 0.001
Heart rate (beats per minute)	191 vs. 263	*P* < 0.001	191 vs. 203	0.090	263 vs. 203	*P* < 0.001
Respiratory rate (breath per minute)	52 vs. 39	*P* < 0.001	52 vs. 46	0.070	39 vs. 46	0.074
SAP (mmHg)	120 vs. 147	*P* < 0.001	120 vs. 140	0.009**	147 vs. 140	0.326
Vertebral heart size	7.6 vs. 7.6	1.000	7.6 vs. 7.7	1.000	7.6 vs. 7.7	1.000
LVFS (%)	49.4 vs. 50.7	0.782	49.4 vs. 51.1	0.403	50.7 vs. 51.1	1.000
LVWT (mm)	4.38 vs. 4.78	0.003**	4.38 vs. 4.40	0.055	4.78 vs. 4.4	0.581
LAFS (%)	42.5 vs. 46.9	0.258	42.5 vs. 43.8	1.000	46.9 vs. 43.8	0.677
LA/Ao	1.31 vs. 1.31	1.000	1.31 vs. 1.32	1.000	1.3 vs. 1.32	1.000
LAD (mm)	11.9 vs. 12.0	1.000	11.9 vs. 11.9	1.000	12.0 vs. 11.9	1.000

Values are presented as mean for parametric data or median for non-parametric data. SAP, systolic arterial blood pressure; LVFS, left ventricular fractional shortening; LVWT, left ventricular wall thickness; LAFS, left atrial fractional shortening; LAD, left atrial diameter; pre, preoperation; 0 h post, 0 h postoperation; 18 h post, 18 h postoperation. Body temperature, heart rate, respiratory rate, vertebral heart size, LVFS, LAFS, LA/Ao, and LAD are normally distributed (parametric data). SAP and LVWT are not normally distributed (non-parametric data). Asterisk indicates statistical difference (**P < 0.01).

**Figure 2:**
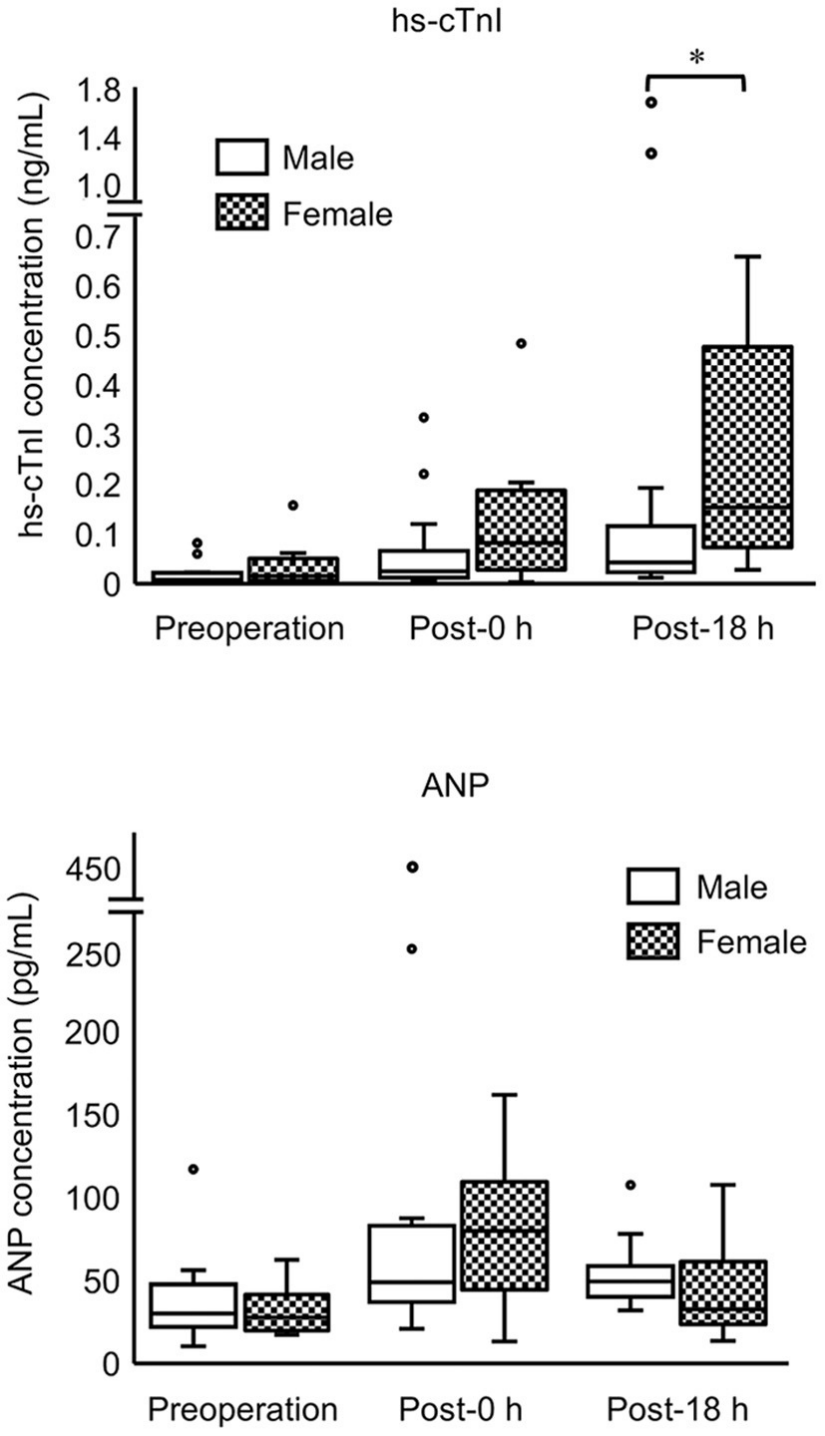
Concentrations of hs-cTnI and ANP in male and female cats at three measurement points (preoperation, 0 h postoperation, 18 h postoperation). The top and bottom of the box represent the 75th and 25th percentiles, respectively; middle line, median; whiskers, highest and lowest data points within 1.5 times the length of the quartiles; and circles, outliers. Data of hs-cTnI and ANP are not normally distributed (non-parametric data). Asterisks indicate statistical difference (*P < 0.05).

Comparison between the male (*n* = 15) and female (*n* = 15) groups showed that hs-cTnI levels at post-18 h and anesthetic time were significantly higher in females than in males (Figure 2; Table 3). Moreover, LVWT levels at three measurement points and BW were significantly higher in males than in females (Tables 1, 3). No significant difference was observed between males and females in ANP or other variables (Figure 2; Table 3).

**Table 3:** Comparison of echocardiographical measurement and clinical data between males and females at preoperation, 0 h postoperation, and 18 h postoperation.

**Variables**	**Preoperation**		**0 h postoperation**		**18 h postoperation**	
	**Value**	***P*-value**	**Value**	***P*-value**	**Value**	***P*-value**
Body temperature (°C)	38.5 vs. 38.2	0.370	37.5 vs. 37.5	0.771	38.4 vs. 38.6	0.868
Heart rate (beats per minute)	184 vs. 196	0.604	260 vs. 264	0.507	183 vs. 210	0.290
Respiratory rate (breath per minute)	50 vs. 55	0.070	39 vs. 39	0.834	48 vs. 43	0.108
SAP (mmHg)	122 vs. 119	1.000	148 vs. 141	0.309	142 vs. 139	0.983
Vertebral heart size	7.6 vs. 7.6	0.590	7.6 vs. 7.6	0.893	7.6 vs. 7.7	0.256
LVFS (%)	49.8 vs. 49.0	0.653	51.2 vs. 49.5	0.204	51.9 vs. 50.3	0.364
LVWT (mm)	4.6 vs. 4.2	*P* < 0.001	5.1 vs. 4.3	*P* < 0.001	4.9 vs. 4.3	*P* < 0.001
LAFS (%)	41.9 vs. 43.1	0.590	46.1 vs. 47.7	0.475	43.1 vs. 44.6	0.499
LA/Ao	1.34 vs. 1.3	0.170	1.32 vs. 1.34	0.884	1.31 vs. 1.34	0.983
LAD (mm)	11.9 vs. 11.8	0.780	11.9 vs. 12.2	0.444	12.0 vs. 11.8	0.404

Values are shown as male vs. female and presented as mean for parametric data or median for non-parametric data. SAP, systolic arterial blood pressure; LVFS, left ventricular fractional shortening; LVWT, left ventricular wall thickness; LAFS, left atrial fractional shortening; LAD, left atrial diameter. Respiratory rate, vertebral heart size, LVFS, LVWT, LAFS, and LAD are normally distributed (parametric data). Body temperature, heart rate, SAP, and LA/Ao are not normally distributed (non-parametric data).

We examined the correlations among the variables with significance in the above statistical analyses (Table 4). Correlation data among all variables for all cats are shown in Supplementary Tables 1–3. Particularly, in all groups, significant positive correlations were found as follows: hs-cTnI vs. HR, ANP vs. HR, ANP vs. SAP, HR vs. SAP, and HR vs. LVWT from pre to post-0 h and hs-cTnI vs. SAP, and HR vs. LVWT from pre to post-18 h (Table 4). By contrast, no correlation was found in all groups as follows: hs-cTnI vs. ANP, hs-cTnI vs. SAP, hs-cTnI vs. LVWT, and ANP vs. LVWT from pre to post-0 h and hs-cTnI vs. ANP, hs-cTnI vs. HR, hs-cTnI vs. LVWT, ANP vs. HR, ANP vs. SAP, ANP vs. LVWT, and HR vs. SAP from pre to post-18 h (Table 4). Moreover, significant positive correlations were noted between hs-cTnI and anesthetic time at post-0 h and post-18 h in females and between BW and LVWT at three measurement points in males (Table 5).

**Table 4:** Correlation coefficients for hs-cTnI, ANP, HR, SAP, and LVWT in all cats.

	**All cats from**		**All cats from**	
	**preoperation to 0 h**		**preoperation to 18 h**	
	**postoperation**		**postoperation**	
	***r***	***P*-value**	***r***	***P-*value**
hs-cTnI vs. ANP	−0.052	0.712	0.161	0.246
hs-cTnI vs. HR	0.303	0.019*	0.217	0.096
hs-cTnI vs. SAP	0.229	0.079	0.354	0.006**
hs-cTnI vs. LVWT	−0.009	0.949	0.052	0.700
ANP vs. HR	0.391	0.004**	0.101	0.468
ANP vs. SAP	0.427	0.001**	0.042	0.765
ANP vs. LVWT	0.087	0.530	0.138	0.321
HR vs. SAP	0.559	*P* < 0.001	0.064	0.628
HR vs. LVWT	0.441	*P* < 0.001	0.327	0.011*

Each data represents the value of Spearman's *r* and its *P-*value. hs-cTnI, high-sensitive cardiac troponin I; ANP, atrial natriuretic peptide; SAP, systolic arterial blood pressure; HR, heart rate; LVWT, left ventricular wall thickness. Asterisk indicates statistical differences (*P < 0.05, **P < 0.01).

**Table 5:** Correlation coefficients for hs-cTnI, anesthetic time, and body weight in females, and between body weight and LVWT in males.

	***R***	***P*-value**
Female		
Anesthetic time vs. body weight	−0.345	0.062
At preoperation		
hs-cTnI vs. anesthetic time	0.309	0.096
hs-cTnI vs. body weight	0.022	0.909
At 0 h postoperation		
hs-cTnI vs. anesthetic time	0.418	0.023*
hs-cTnI vs. body weight	0.286	0.125
At 18 h postoperation		
hs-cTnI vs. anesthetic time	0.386	0.035*
hs-cTnI vs. body weight	0.182	0.337
Male		
At preoperation		
Body weight vs. LVWT	0.7855	*P* < 0.001
At 0 h postoperation		
Body weight vs. LVWT	0.8435	*P* < 0.001
At 18 h postoperation		
Body weight vs. LVWT	0.9696	*P* < 0.001

Each data represents the value of Spearman's *r* and its *P*-value. hs-cTnI, high-sensitive cardiac troponin I; LVWT, left ventricular wall thickness. Asterisk indicates statistical differences (*P < 0.05).

## Discussion

In this study, we describe the perioperative changes in hs-cTnI and ANP with cardiac parameters in juvenile cats. All cats had increased postoperative hs-cTnI compared with the presurgical levels. In particular, 48% of cats (14/29) experienced elevated hs-cTnI over RI after surgery. In humans, 17.9% of patients in non-cardiac surgery have elevated hs-cardiac troponin over a reference range in perioperation (4). By contrast, 6.7% of dogs (two out of 30) under general anesthesia have increased cTnI concentration over the upper limit of normal in conventional cTnI assay after anesthesia compared with preanesthesia (15). Verbiest et al. reported elevated hs-cTnI concentration in 55% of dogs after general anesthesia compared with the corresponding preoperative values, although a specific reference range was unavailable in their study (5). Considering the varied cardiac troponin levels between species, postoperative hs-cTnI levels might increase in juvenile cats more easily than in humans or dogs; this finding might contribute to elucidating why TMT often occurs in juvenile cats.

Moreover, HR at post-0 h and SAP at post-0 h and post-18 h were significantly increased compared with those at pre in all groups in this study, whereas RR and BT were significantly decreased in the same manner. Immediately after surgery in cats, HR and SAP normally increase and RR and BT decrease compared to their respective basal values at preoperation (16, 17). Furthermore, the elevated SAP after surgery gradually decreases and stabilizes after 10 days in cats (18). A significant positive correlation was found between hs-cTnI and SAP at post-18 h in our study. In humans, SAP is associated with hs-cTnI level due to the increase in cardiac afterload (19). However, the pathophysiology of MINS is not only attributed to elevated SAP but also multifactorial; MINS is divided into plaque rupture (type-1 MI) or myocardial oxygen supply-demand imbalance (type-2 MI) (1, 2). Assuming that MINS can occur in cats, the perioperative increase in hs-cTnI may be attributed to type-2 MI due to the rare occurrence of atherosclerosis in cats (12). Perioperative triggers for myocardial ischemia or infarction related to type-2 MI include perioperative inflammation, stress response, and hemodynamic changes by activating sympathetic tone (20). In particular, surgical stress response promotes a higher level of inflammation and sympathetic activation resulting in significant hemodynamic changes in cats (21, 22). Thus, considering the result of increased hs-cTnI and hemodynamic changes, surgical stress and subsequent hemodynamic changes can greatly promote PMI in cats.

In our study, hs-cTnI levels were higher in females than in males after surgery. This finding is not surprising because spaying takes longer and is more invasive than castration. Anesthetic time was longer in females than in males, and a significant positive correlation was found between hs-cTnI and anesthetic time. Moreover, we used cardio-protective drugs in this study; isoflurane and buprenorphine reduce myocardial damage (23, 24). Despite this, myocardial damage was detected in many cats in our research, suggesting that lessening the duration of anesthesia is better and further study is warranted to alleviate PMI in cats. Cats tend to experience anxiety at the hospital, and temporary hemodynamic changes are often observed. Thus, further studies to evaluate the effects of stress-reducing drugs, such as gabapentin and/or other analgesics, for feline PMI are needed.

The levels of ANP and LVWT significantly increased at post-0 h compared with pre-operative levels. Moreover, ANP was positively correlated with HR and SAP, and LVWT was positively correlated with HR. Although elevated ANP indicates enlarged left atrial size, the rise in SAP may increase ANP as a false-positive in cats (25). Similarly, LVWT can be thicker as HR increases in cats (26). Incidentally, LVWT was positively correlated with BW in males, consistent with a previous report (27). No correlation was found between hs-cTnI and LVWT or between ANP and left atrial size, including LA/Ao, LA/FS, and LAD in our study. Therefore, ANP might have increased independently of hs-cTnI, and PMI might not affect the myocardial thickness for a brief time.

When acute myocardial infarction occurs, N-terminal-pro brain natriuretic peptide (NT-proBNP) as well as cardiac troponin T (cTnT) also increases in humans (28). However, the peak time in cTnT is faster than in NT-proBNP in patients with acute myocardial infarction (28). Moreover, not NT-proBNP but cardiac troponin assay is used in the diagnosis of acute myocardial injury in humans because cardiac troponin directly reflects myocardial injury. Especially, measuring hs-cTn can detect slight myocardial injury in the acute phase (2, 3). Instead, NT-proBNP would be useful to evaluate prognosis and cardiac risk in non-cardiac surgery (29). Generally, ANP is not used in evaluation for PMI, while in cats there is no research on perioperative changes in ANP in cats. Thus, we chose ANP and hs-cTnI in this study to evaluate acute changes in cardiac biomarkers in a short term within 18 h.

After myocardial infarction, the increase in cardiac troponin can be detected within 2–4 h, and peak concentration is frequently reached in 18–24 h (12). In this study, there was a time limitation in that some owners hoped for their cats to be discharged the next morning (from 09:00). We had to collect samples at post-0 h in the afternoon (from 14:00 to 15:00), so we needed to collect samples at post-18 h before discharge time (before 09:00). Thus, we set the last sampling time at 18 h.

Diurnal variation of cardiac troponin has been reported in humans (30). The samples for all cats in our study were collected preoperatively in the morning from 09:00 to 12:00, at post-0 h in the afternoon (from 14:00 to 15:00), and at post-18 h in the next morning (from 08:00 to 09:00) when 18 h passed from post-0h. According to Fournier et al., the nadir value of high-sensitive cTnT was observed at 18:00, while the peak value was observed at 06:00 (30). Although no research on circadian fluctuation of cTn was available for dogs or cats, considering the fact that the levels of hs-cTnI were significantly higher at post-0 h (evening) and post-18 h (morning) than at pre (morning), the influence of diurnal fluctuation to the increase hs-cTnI would have been minimal. Some natriuretic peptides, including NT-proBNP have circadian fluctuation, while several studies reported that midregional pro-ANP or ANP did not show the diurnal fluctuation (31, 32). Although no research on circadian fluctuation of ANP was available for dogs or cats, ANP would not be affected by circadian rhythm in cats.

This study had several limitations. First, measurement of cardiac biomarkers at the recovery point was unavailable because the hospitalization duration was short, and permission could not be obtained from the owners. Fortunately, no cat exhibited any cardiac clinical signs at least 1 week after surgery, but the diagnostic criteria of MINS in cats and whether the elevated hs-cTnI is reversible or irreversible remain uncertain. Thus, further long-term observation in the change in perioperative hs-cTnI is necessary to elucidate the pathophysiology of TMT or criteria and prognosis of MINS, which might be related to a higher mortality rate at perianesthesia in cats than in dogs (33).

Second, interpretation of the results of several statistical analyses was limited due to the rather small sample. However, statistically significant differences among three measuring points in hs-cTnI, ANP, BT, HR, RR, SAP, VHS, LVFS, LVWT, and LAFS in all cats and between males and females in BW and anesthetic time were detected with at least 80% power in this small pilot study. Likewise, significant correlations with at least 80% power were found as follows: ANP vs. HR, ANP vs. SAP, HR vs. SAP, and HR vs. LVWT from pre to post-0 h and hs-cTnI vs. SAP from pre to post-18 h. Several pilot studies related to perioperative change in cTnI in dogs demonstrated significant results with relatively small samples of *N* = 18 to *N* = 33 (5, 15, 34), and in one of these studies, the robustness of a significant difference was confirmed statistically by a power analysis to confirm whether the sample size was adequate or not (15). Therefore, the sample size in the current study was adequate to detect significant differences in the above data. However, further studies are needed to investigate the difference between males and females in PMI due to the rather low statistical power to detect significant differences between males and females in this study.

Third, confounding factors that increase hs-cTnI might have been present; oxidative stress, tachycardia, inotropic or vasopressor drug, and BW can cause higher cardiac troponin (11, 35). In this study, all cats (30/30) experienced increased postoperative hs-cTnI compared with the presurgical levels including slight changes in hs-cTnI. We used high-sensitive cTnI assay with a lower of limit (0.006 ng/mL), and this assay can detect slight changes in cTnI. Atropine was used in this study, leading to the increase in myocardial oxygen consumption due to increased HR (36). Thus, this small change in hs-cTnI might be attributed to factors, such as using atropine. Body weight was significantly lower in females than in males in this study, while no correlation was found between hs-cTnI and body weight at the three measuring points. Moreover, no correlation was found between anesthetic time and body weight in females. Since the level of hs-cTnI at post-18 h and anesthetic time were significantly higher in females than in males and no significant differences were found in the level of hs-cTnI at pre and post-0 h, body weight does not seem to affect the increase in hs-cTnI in females. Considering the fact that 48% of cats and females, which had lesser BW than males, experienced greatly elevated hs-cTnI over reference range; these confounding factors seem to have been less influential to the increase in hs-cTnI over a reference interval in 48% of cats in our study.

## Conclusions

All cats showed increased hs-cTnI after operation compared with preoperation, and 48% of cats experienced elevated hs-cTnI over reference range after surgery. The myocardium in juvenile cats may be vulnerable to surgical stress due to the higher possibility of MINS occurrence. Measuring hs-cTnI at perioperation would be beneficial for early detection and evaluation of PMI presence in cats. To evaluate the risk of MINS and the relationship between PMI and TMT, further research about MINS in cats is warranted.

## Data availability statement

The original contributions presented in the study are included in the article/Supplementary material, further inquiries can be directed to the corresponding author/s.

## Ethics statement

The animal study was reviewed and approved by the Ethics Committee for the Use of Animals in Research of Yokohama Animal Medical Center Kannai Animal Clinic, Yokohama, Kanagawa, Japan (Approval number: 2021-01; Approval date: September 15, 2021). Written informed consent was obtained from the owners for the participation of their animals in this study.

## Author contributions

KK: sample collection, data analysis, and writing the draft. MS and CS: data analysis for cardiac biomarkers. SO, SK, TN, HT, and TU: sample collection and data analysis. MI: study design and data analysis. All authors contributed to the article and approved the submitted version.

## Conflict of interest

High-sensitive cardiac troponin I and ANP analyses were performed free of charge by FUJIFILM VET Systems Co., Ltd., Japan. Authors MS and CS are affiliated with FUJIFILM VET Systems Co., Ltd. The remaining authors declare that the research was conducted in the absence of any commercial or financial relationships that could be construed as a potential conflict of interest.

## Publisher's note

All claims expressed in this article are solely those of the authors and do not necessarily represent those of their affiliated organizations, or those of the publisher, the editors and the reviewers. Any product that may be evaluated in this article, or claim that may be made by its manufacturer, is not guaranteed or endorsed by the publisher.

## Abbreviations

ANP: atrial natriuretic peptide
BT: body temperature
BW: body weight
cTnI: cardiac troponin I
cTnT: cardiac troponin T
DLVOTO: dynamic left ventricular outflow tract obstruction
ECG: electrocardiogram
FIP: feline infectious peritonitis
HCM: hypertrophic myocardiopathy
HR: heart rate
hs-cT: high-sensitive cardiac troponin
hs-cTnI: high-sensitive cardiac troponin I
IV: intravenous injection
LA/Ao: left atrium to aorta ratio
LAD: left atrial diameter
LAFS: left atrial fractional shortening
LVFS: left ventricular fractional shortening
LVWT: left ventricular wall thickness
MINS: myocardial injury after non-cardiac surgery
NT-proBNP: N-terminal-pro brain natriuretic peptide
PMI: perioperative myocardial injury
RI: reference interval
RPLA: right parasternal long-axis
RPSA: right parasternal short-axis view
RR: respiratory rate
SAM: systolic anterior motion of the mitral valve
SAP: systolic arterial blood pressure
SC: subcutaneous injection
TMT: transient myocardial thickening
VHS: vertebral heart size.

## References

[1] Puelacher C Lurati-Buse G Singeisen H Dang M Cuculi F Müller C. Perioperative myocardial infarction/injury after noncardiac surgery. Swiss Med Wkly. (2015) 145:w14219. 10.4414/smw.2015.14219 26599804

[2] Iddagoda MT. The role of high-sensitive troponin measurement as a biomarker during the postoperative period for the detection of myocardial injury after non-cardiac surgery. J Periop Pract. (2021) 31:300–5. 10.1177/1750458920930993 32609067

[3] Ruetzler K Smilowitz NR Berger JS Devereaux PJ Maron BA Newby LK. Diagnosis and management of patients with myocardial injury after noncardiac surgery: a scientific statement from the American Heart Association. Circulation. (2021) 144:e287–305. 10.1161/CIR.0000000000001024 34601955

[4] Study Investigators VISION Devereaux PJ. Biccard BM Sigamani A Xavier D Chan MTV Srinathan SK. Association of postoperative high-sensitivity troponin levels with myocardial injury and 30-day mortality among patients undergoing noncardiac surgery. JAMA. (2017) 317:1642–51. 10.1001/jama.2017.4360 28444280

[5] Verbiest T Binst D Waelbers T Coppieters E Polis I. Perioperative changes in cardiac troponin I concentrations in dogs. Res Vet Sci. (2013) 94:4468. 10.1016/j.rvsc.2012.10.023 23178045

[6] Novo Matos J Pereira N Glaus T Wilkie L Borgeat K Loureiro J. Transient myocardial thickening in cats associated with heart failure. J Vet Intern Med. (2018) 32:48–56. 10.1111/jvim.14897 29243322 PMC5787177

[7] Agarwal S Bean MG Hata JS Castresana MR. Perioperative takotsubo cardiomyopathy: a systematic review of published cases. Semin Cardiothorac Vasc Anesth. (2017) 21:277–90. 10.1177/1089253217700511 29098955

[8] Amin HZ Amin LZ Pradipta A. Takotsubo cardiomyopathy: a brief review. J Med Life. (2020) 13:3−7. 10.25122/jml-2018-0067 32341693 PMC7175432

[9] Abbott JA MacLean HN. Two-dimensional echocardiographic assessment of the feline left atrium. J Vet Intern Med. (2006) 20:111–9. 10.1111/j.1939-1676.2006.tb02830.x 16496930

[10] Luis Fuentes V Abbott J Chetboul V Côté E Fox PR Häggström J. consensus statement guidelines for the classification, diagnosis, and management of cardiomyopathies in cats. J Vet Intern Med. (2020) 34:1062–77. 10.1111/jvim.15745 32243654 PMC7255676

[11] Ernandes MA Cantoni AM Armando F Corradi A Ressel L Tamborini A. Feline coronavirus-associated myocarditis in a domestic longhair cat. JFMS Open Rep. (2019) 5:2055116919879256. 10.1177/2055116919879256 31636915 PMC6787879

[12] Langhorn R Willesen JL. Cardiac troponins in dogs and cats. J Vet Intern Med. (2016) 30:36–50. 10.1111/jvim.13801 26681537 PMC4913658

[13] Langhorn R Tarnow I Willesen JL Kjelgaard-Hansen M Skovgaard IM Koch J. Cardiac troponin I and T as prognostic markers in cats with hypertrophic cardiomyopathy. J Vet Intern Med. (2014) 28:1485–91. 10.1111/jvim.12407 25056593 PMC4895561

[14] Lang RM Badano LP Mor-Avi V Afilalo J Armstrong A Ernande L. Recommendations for cardiac chamber quantification by echocardiography in adults: an update from the American Society of Echocardiography and the European Association of Cardiovascular Imaging. J Am Soc Echocardiogr. (2015) 28:1–39.e14. 10.1016/j.echo.2014.10.003 25559473

[15] Saunders AB Hanzlicek AS Martinez EA Stickney MJ Steiner JM Suchodolski JS. Assessment of cardiac troponin I and C-reactive protein concentrations associated with anesthetic protocols using sevoflurane or a combination of fentanyl, midazolam, and sevoflurane in dogs. Vet Anaesth Analg. (2009) 36:449–56. 10.1111/j.1467-2995.2009.00483.x 19709049

[16] Brondani JT Loureiro Luna SP Beier SL Minto BW Padovani CR. Analgesic efficacy of perioperative use of vedaprofen, tramadol or their combination in cats undergoing ovariohysterectomy. J Feline Med Surg. (2009) 11:4209. 10.1016/j.jfms.2008.10.002 19233698 PMC10832834

[17] Beths T Touzot-Jourde G Musk G Pasloske K. Clinical evaluation of alfaxalone to induce and maintain anaesthesia in cats undergoing neutering procedures. J Feline Med Surg. (2014) 16:609–15. 10.1177/1098612X13514420 24305470 PMC11164152

[18] Mishina M Watanabe N Watanabe T. Diurnal variations of blood pressure in cats. J Vet Med Sci. (2006) 68:243–8. 10.1292/jvms.68.243 16598167

[19] Aeschbacher S Schoen T Bossard M van der Lely S Glättli K Todd J. Relationship between high-sensitivity cardiac troponin I and blood pressure among young and healthy adults. Am J Hypertens. (2015) 28:789–96. 10.1093/ajh/hpu226 25424717

[20] Smit M Coetzee AR Lochner A. The pathophysiology of myocardial ischemia and perioperative myocardial infarction. J Cardiothorac Vasc Anesth. (2020) 34:2501–12. 10.1053/j.jvca.2019.10.005 31685419

[21] Moldal ER Kirpensteijn J Kristensen AT Haga HA Nødtvedt A Eriksen T. Evaluation of inflammatory and hemostatic surgical stress responses in male cats after castration under general anesthesia with or without local anesthesia. Am J Vet Res. (2012) 73:1824–31. 10.2460/ajvr.73.11.1824 23106471

[22] Moldal ER Eriksen T Kirpensteijn J Nødtvedt A Kristensen AT Sparta FM. Intratesticular and subcutaneous lidocaine alters the intraoperative haemodynamic responses and heart rate variability in male cats undergoing castration. Vet Anaesth Analg. (2013) 40:63–73. 10.1111/j.1467-2995.2012.00773.x 23033908

[23] Kato R Foëx P. Myocardial protection by anesthetic agents against ischemia-reperfusion injury: an update for anesthesiologists. Can J Anaesth. (2002) 49:777–91. 10.1007/BF03017409 12374705

[24] Tempe DK Dutta D Garg M Minhas H Tomar A Virmani S. Myocardial protection with isoflurane during off-pump coronary artery bypass grafting: a randomized trial. J Cardiothorac Vasc Anesth. (2011) 25:59–65. 10.1053/j.jvca.2010.03.002 20580572

[25] Lalor SM Connolly DJ Elliott J Syme HM. Plasma concentrations of natriuretic peptides in normal cats and normotensive and hypertensive cats with chronic kidney disease. J Vet Cardiol. (2009) 11:S71–9. 10.1016/j.jvc.2009.01.004 19398225

[26] Sugimoto K Fujii Y Ogura Y Sunahara H Aoki T. Influence of alterations in heart rate on left ventricular echocardiographic measurements in healthy cats. J Feline Med Surg. (2017) 19:841–5. 10.1177/1098612X16661374 27502088 PMC11104112

[27] Häggström J Andersson ÅO Falk T Nilsfors L OIsson U Kresken JG. Effect of body weight on echocardiographic measurements in 19,866 pure-bred cats with or without heart disease. J Vet Int Med. (2016) 30:1601–11. 10.1111/jvim.14569 27573384 PMC5032876

[28] Gill D Seidler T Troughton RW Yandle TG Frampton CM Richards M. Vigorous response in plasma N-terminal pro-brain natriuretic peptide (NT-BNP) to acute myocardial infarction. Clin Sci. (2004) 106:135–9. 10.1042/CS20030131 12974669

[29] Faloye AO Gebre MA Bechtel AJ. Predicting cardiac risk in noncardiac surgery: a narrative review. J Anesth. (2021) 35:122–9. 10.1007/s00540-020-02868-7 33141342

[30] Fournier S Iten L Marques-Vidal P Boulat O Bardy D Beggah A. Circadian rhythm of blood cardiac troponin T concentration. Clin Res Cardiol. (2017) 106:1026–32. 10.1007/s00392-017-1152-8 28856443

[31] Breidthardt T van Doorn WPTM van der Linden N Diebold M Wussler D Danier I. Diurnal variations in natriuretic peptide levels: clinical implications for the diagnosis of acute heart failure. Circ Heart Fail. (2022) 15:e009165. 10.1161/CIRCHEARTFAILURE.121.009165 35670217 PMC10004748

[32] Chiang FT Tseng CD Hsu KL Lo HM Tseng YZ Hsieh PS. Circadian variations of atrial natriuretic peptide in normal people and its relationship to arterial blood pressure, plasma renin activity and aldosterone level. Int J Cardiol. (1994) 46:229–33. 10.1016/0167-5273(94)90245-3 7814177

[33] Matthews NS Mohn TJ Yang M Spofford N Marsh A Faunt K. Factors associated with anesthetic-related death in dogs and cats in primary care veterinary hospitals. J Am Vet Med Assoc. (2017) 250:655–65. 10.2460/javma.250.6.655 28263113

[34] Planellas M Cuenca R Tabar MD Bertolani C Poncet C Closa JM. Clinical assessment and C-reactive protein (CRP), haptoglobin (Hp), and cardiac troponin I (cTnI) values of brachycephalic dogs with upper airway obstruction before and after surgery. Can J Vet Res. (2015) 79:58–63. Available online at: https://www.canadianveterinarians.net/journals-and-classified-ads/canadian-journal-of-veterinary-research/25673910 PMC4283235

[35] Hori Y Iguchi M Heishima Y Yamashita Y Nakamura K Hirakawa A. Diagnostic utility of cardiac troponin I in cats with hypertrophic cardiomyopathy. J Vet Intern Med. (2018) 32:922–9. 10.1111/jvim.15131 29660794 PMC5980312

[36] Huang HY Liao KY Shia WY Chang CC Wang HC. Effect of administering dexmedetomidine with or without atropine on cardiac troponin I level in isoflurane-anesthetized dogs. J Vet Med Sci. (2021) 83:1869–76. 10.1292/jvms.20-0657 34629333 PMC8762405

